# ASO Author Reflections: How Does Emphysema Contribute to Poor Prognosis After Lung Cancer Surgery?

**DOI:** 10.1245/s10434-024-15171-6

**Published:** 2024-05-08

**Authors:** Masayuki Ishida, Takahiro Mimae, Morihito Okada

**Affiliations:** Department of Surgical Oncology, Hiroshima University, Hiroshima, Japan

## Past

Emphysema is considered a poor prognostic factor in patients with non-small cell lung cancer. Emphysema often coexists with primary lung cancer,^1^ and lung cancer arising from emphysema is often highly malignant.^2^ However, whether the poor prognosis of patients with lung cancer and emphysema is caused by the emphysema itself or the high-grade malignancy of lung cancer, which is observed more frequently in emphysematous lungs than in healthy lungs, remains unknown.

## Present

This study aimed to clarify the prognostic impact of emphysema in patients with early non-small cell lung cancer. Our results demonstrated that patients with emphysema had a poorer 5-years overall survival rate, were more likely to develop highly malignant tumors, and had a higher cumulative incidence of other causes of death than those with healthy lungs. The risk of other causes of death was higher in patients with both mildly and severely emphysematous lungs. In patients with early non-small cell lung cancer, emphysema contributed to poor prognosis after complete pulmonary resection, not only through the development of highly malignant tumors but also by increasing the risk of other causes of death that are attributable to emphysema.^3^

## Future

Although the results of this study need to be validated in larger cohorts, in patients with mild emphysema, both high-grade lung cancer malignancy and the risk of other causes of death should be considered while determining appropriate treatment strategies.
